# Supplementary material for: Genome-wide association studies from spoken phenotypic descriptions: a proof of concept from maize field studies
Source: G3 (Bethesda). 2024 Aug 5;14(9):jkae161. doi: 10.1093/g3journal/jkae161 (PMC11373645; doi:10.1093/g3journal/jkae161)
Supplement: jkae161_Supplementary_Data [file jkae161_supplementary_data.zip › Supplemental_Figure_1_G3-2024-405122.pdf]

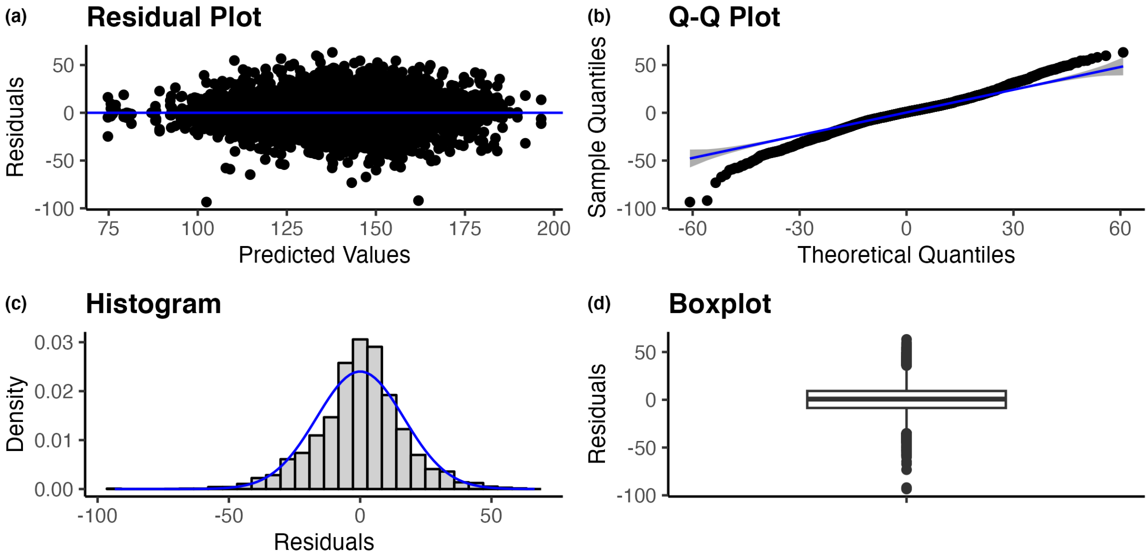

Supplementary Figure 1 Diagnostic plots for the linear regression model used for BLUEs estimation for measured plant height data. (a) Residual plot of the residuals versus the predicted values of the model, (b) Q−Q Plot of sample vs theoretical quantiles, (c) Histogram of residuals with normal density curve overlayed, (d) Boxplot of residuals.
