# Supplementary material for: Genome-wide association studies from spoken phenotypic descriptions: a proof of concept from maize field studies
Source: G3 (Bethesda). 2024 Aug 5;14(9):jkae161. doi: 10.1093/g3journal/jkae161 (PMC11373645; doi:10.1093/g3journal/jkae161)
Supplement: jkae161_Supplementary_Data [file jkae161_supplementary_data.zip › Supplemental_Figure_7_G3-2024-405122.pdf]

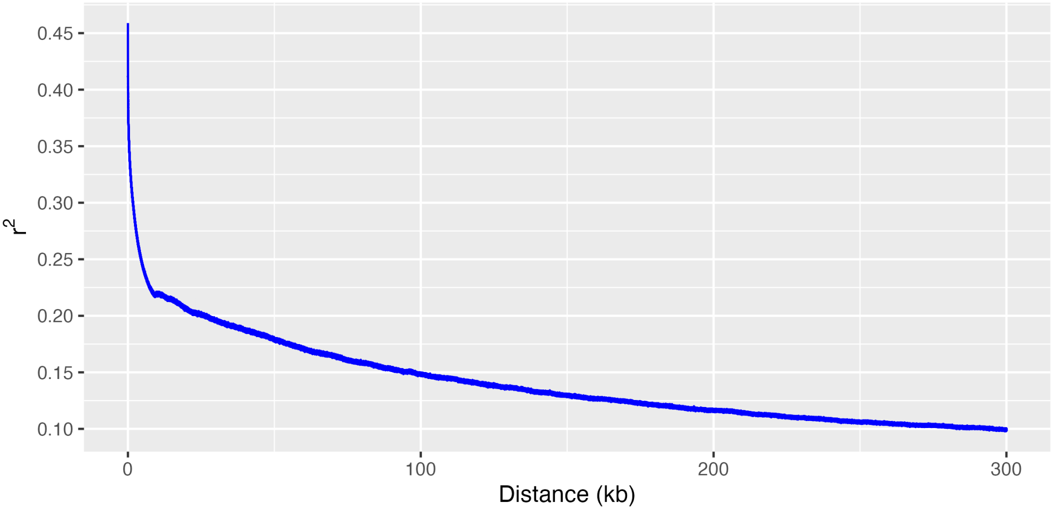

Supplementary Figure 7 LD decay curve using Mural et al. data taxa intersecting with Yanarella et al. taxa. Blue line represents the plot of r^2 vs distance in kb.
